# A Novel Therapy for Cisplatin-Induced Allodynia and Dysfunctional and Emotional Impairments in Male and Female Mice

**DOI:** 10.3390/antiox12122063

**Published:** 2023-11-30

**Authors:** Ignacio Martínez-Martel, Olga Pol

**Affiliations:** 1 Grup de Neurofarmacologia Molecular, Institut de Recerca Sant Pau, Sant Quintí 77-79, 08041 Barcelona, Spain; 2 Grup de Neurofarmacologia Molecular, Institut de Neurociències, Universitat Autònoma de Barcelona, 08193 Barcelona, Spain

**Keywords:** anxiety, chemotherapy-induced peripheral neuropathy, cisplatin, depression, hydrogen-rich water, inflammation, molecular hydrogen, oxidative stress

## Abstract

Patients undergoing chemotherapy with cisplatin (CIS) develop neuropathy in addition to other symptoms such as, anxiety, depression, muscle wasting and body weight loss. This symptomatology greatly weakens patients and may even lead to adjournment of chemotherapy. The protecting actions of molecular hydrogen in many neurological illnesses have been described, but its effect on the functional and emotional deficiencies caused by CIS has not been assessed. In C57BL/6J male and female mice injected with CIS, we examined the impact of the prophylactic treatment with hydrogen-rich water (HRW) on: (i) the tactile and cold allodynia, (ii) the deficits of grip strength and weight loss, (iii) the anxiodepressive-like behaviors and (iv) the inflammatory and oxidative reactions incited by CIS in the dorsal root ganglia (DRG) and prefrontal cortex (PFC). The results demonstrate that the mechanical allodynia and the anxiodepressive-like comportment provoked by CIS were similarly manifested in both sexes, whereas the cold allodynia, grip strength deficits and body weight loss produced by this chemotherapeutic agent were greater in female mice. Nonetheless, the prophylactic treatment with HRW prevented the allodynia and the functional and emotional impairments resulting from CIS in both sexes. This treatment also inhibited the inflammatory and oxidative responses activated by CIS in the DRG and PFC in both sexes, which might explain the therapeutic actions of HRW in male and female mice. In conclusion, this study revealed the plausible use of HRW as a new therapy for the allodynia and physical and mental impairments linked with CIS and its possible mechanism of action.

## 1. Introduction

Chemotherapy-associated neuropathic pain is a major unpleasant effect resulting from this treatment in cancer patients. Patients undergoing chemotherapy also develop other signs, such as anxiety, depression, muscle wasting and cognitive deficits that have a significant negative impact on the well-being and quality of life of patients and may even lead to the need for adjournment of the chemotherapy [1,2]. Furthermore, therapies to prevent or treat this type of pain such as anticonvulsant, antidepressant, and non-steroidal anti-inflammatory drugs are not significantly effective and produce noteworthy side effects [3,4]. Therefore, our objective is to find an effective and safe treatment for alleviating the neuropathic pain related to chemotherapy, as well as the functional deficits and mood disorders that are associated with it.

Cisplatin (CIS), a platinum analogue discovered by Rosenberg et al. (1969) [5], remains one of the most used chemotherapeutic agents to treat solid tumors located in different organs, including the ovaries, testes, head, neck, breast and lung [6,7]. However, despite the demonstrable clinical advantages of CIS as an antineoplastic drug, this treatment entails neuropathic pain and affective deficits, as well as muscle wasting and body weight loss, which can persist for months and greatly weaken patients [8,9,10]. All the side effects caused by CIS chemotherapy are relevant since they are the main reasons for a clinical decline in cancer patients [10]. Thus, effective treatments dismissing CIS-caused adverse effects are necessary.

It is accepted that gender differences exist in the pathology of neuropathic pain. Several clinical studies have demonstrated that women are more sensitive to pain than men [11,12]. Additionally, animal investigations have also reported that female rodents show a lower threshold for pain in different models [13]. Regarding the impact of sex in the chemotherapy-induced neuropathic pain provoked by CIS, contradictory results exist. Some studies revealed that the mechanical and/or cold allodynia caused by CIS are similarly displayed in male and female animals [4,14], whereas another showed sex discrepancies in the maintenance of mechanical allodynia [15]. Nevertheless, possible differences among sexes in the development of CIS-induced emotional disorders, muscle wasting and/or weight lose have been poorly investigated.

Treatment with CIS activates the transcription factor NF-κB and the successive pro-inflammatory cytokines (TNF-α, IL-6 and IL-8), leading to neuronal inflammation [16,17]. These inflammatory mediators play an important role in muscle wasting [18] as well as in the development of the anxiodepressive-like behaviors [19]. It is also well documented that the dysregulation of the NLRP3 inflammasome leads to excessive production of pro-inflammatory cytokines, leading to severe inflammation and/or various diseases such chronic pain [20]. The release of reactive oxygen species (ROS) from the dysfunctional mitochondria is a key upstream action for inducing the activation of the NLRP3 inflammasome [21]. Recent studies have demonstrated that NLRP3 activation drives paclitaxel-, vincristine- and oxaliplatin-induced peripheral neuropathy [22,23,24]. These works further revealed that the inhibition or deletion of NLRP3 prevents the development of neuropathic pain caused by vincristine and oxaliplatin without affecting the chemotherapy efficacy [22,23]. Nonetheless, the role played by this inflammasome in CIS-induced neuropathy is not completely known.

Abundant evidence also indicates that the beginning and progression of chemotherapy-induced neuropathic pain are linked with oxidative stress [25]. Oxidative stress also participates in the CIS-mediated neuropathy through a dysregulation of the products of lipid peroxidation (thiobarbituric acid reactive substance (TBARS) or 4-hydroxy-2-nonenal (4-HNE)) and Nrf2 transcription factor levels, which protect against the free radical injury by regulating antioxidant enzymes, for example heme oxygenase-1 (HO-1), superoxide dismutase-1 (SOD-1) and catalase [26]. Therefore, a decreased catalase and SOD-1 activity in the dorsal root ganglia (DRG) [27] and prefrontal cortex (PFC) of CIS-injected mice [28] was demonstrated. In line with this, increased levels of TBARS were also observed in the PFC of CIS-injected mice [28]. Therefore, the maintenance of a balance between the inflammatory and antioxidant pathways might be key for alleviating CIS-induced neuropathy and associated emotional disorders.

It is acknowledged that CIS-based chemotherapy is also related to other side effects, including muscle wasting and body weight loss [29,30]. In line with this, decreased muscle strength and body weight are manifested in CIS-injected mice [31,32,33]. However, the impact of sex and the possible treatment of these physical dysfunctions produced by CIS requires further investigation.

Molecular hydrogen (H_2_) produces strong anti-inflammatory and antioxidant actions that are predominantly attributed to its capacity to inhibit the synthesis of NF-κB and the subsequent release of pro-inflammatory factors [34,35], as well as by reducing ROS levels and increasing the expression of antioxidant enzymes such as SOD-1 and HO-1 [36]. Recent experiments further revealed that the therapeutic administration of hydrogen-rich water (HRW) has beneficial effects in animals with paclitaxel-induced neuropathic pain [37]. This treatment also reduced the nociceptive and emotive disturbances occasioned by nerve injury and persistent paw inflammation in male mice [38,39]. There is also evidence that H_2_ exhibits antidepressant and/or anxiolytic properties in animals with traumatic brain injury [40] or chronic stress-induced depressive- and anxiety-like comportments [41] and further improves psychiatric illnesses in humans [42]. Nevertheless, it is not known whether the prophylactic treatment with HRW can avoid the CIS-caused mechanical and thermal allodynia and some physical disabilities. Moreover, given that the emotional deficits can have long-lasting effects and negatively modulate pain sensation [43,44], it is also crucial to assess whether HRW administration can effectively inhibit the anxiety- and depressive-like behaviors associated with CIS-induced neuropathy.

Therefore, our study aims to investigate the potential beneficial effects of prophylactic treatment with HRW as a novel approach for controlling CIS-induced neuropathy, weight loss and limb grip strength, as well as the development of anxiodepressive-like behaviors. The impact of sex on these actions and the main pathways involved will also be studied.

## 2. Materials and Methods

### 2.1. Animals

Male and female C57BL/6J mice at 10 weeks of age at the beginning of the experiments were used in this study. Mice acquired from Envigo Laboratories (Barcelona, Spain) were housed with a 12 h dark/light cycle, 22 °C temperature and 66% humidity with food and water ad libitum. After 7 days of acclimatization to the housing conditions, the experimental procedure was conducted between 9:00 to 17:00 h in agreement with the guidelines of the European Commission’s directive (2010/63/EC) and the Spanish Law (RD 53/2013) regulating animal research; the experiments were approved by the local Committee of Animal Use and Care of the Autonomous University of Barcelona (CEEA-UAB-protocol # 4581). A total of 128 mice (64 male and 64 female) were used in this study. Mice were randomly assigned to experimental groups. Animals were housed in polypropylene cages with four male or female mice per cage in an enriched environment that included a carton hut and cellulose fragments. The experiments were performed by a researcher blinded to the experimental conditions. All efforts were made to minimize animal suffering and to reduce the number of animals used.

### 2.2. Experimental Design

In different groups of male and female mice:

(1) The development of tactile and cold allodynia, deficits of grip strength and body weight loss provoked by the intraperitoneal administration of CIS or VEH were evaluated on days 2, 4, 6, 9, 11, 13, 16, 18, 20, 23, 25, 27 and 30 after their first injection (*n* = 8 animals). 

(2) The anxiety- and/or depressive-like behaviors caused by CIS or VEH were evaluated at day 30 after their first injection (*n* = 8 animals).

(3) The inhibitory actions of co-treatment with HRW (from day 0 to 30) on the tactile and cold allodynia, grip strength deficits and body weight loss caused by CIS or VEH were evaluated on days 2, 4, 6, 9, 11, 13, 16, 18, 20, 23, 25, 27 and 30 after the first injection of CIS or VEH (*n* = 8 animals).

(4) The possible anxiolytic and antidepressant effects produced by the co-treatment with HRW were evaluated at day 30 after the first administration of CIS or VEH (*n* = 8 animals).

(5) The effects of HRW on the protein levels of NLRP3, 4-HNE, HO-1 and SOD-1 in the DRG and PFC were evaluated at day 30 after the first administration of CIS or VEH using Western blot (*n* = 3 samples for group). 

**Figure 1:**
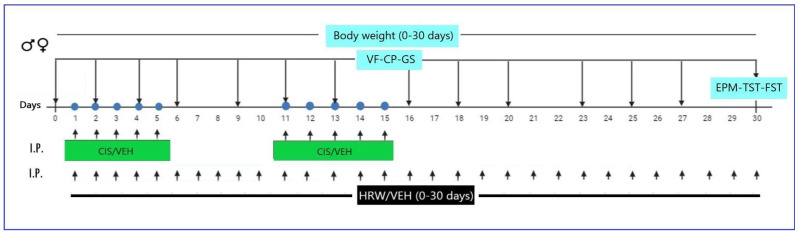
Schematic representation of the experimental timeline. VF, von Frey; CP, cold plate; GS, grip strength; EPM, elevated plus maze; TST, tail suspension test; FST, forced swimming test; I.P., intraperitoneal; CIS, cisplatin; VEH, vehicle; HRW, hydrogen-rich water.

The experimental design is represented in Figure 1.

### 2.3. Drug Administration

CIS (Sigma Aldrich, St. Louis, MO, USA) was diluted in sterile saline (0.9% NaCl) and given intraperitoneally daily at a dosage of 2.8 mg/kg for 5 days, followed by 5 days of rest, and as second round of 5 doses, according to a modified version reported in [45,46]. The doses and administration regimen of CIS were chosen as they were similar to those used in patients undergoing this type of chemotherapy [47]. HRW was prepared using a hydrogen water generator from hydrogen (Osmo-star Soriano S.L., Alicante, Spain) according to Cheng et al. (2020) [48]. All drugs were newly prepared before their administration. For each group treated with a drug, the respective control group received the same volume of analogous vehicle (VEH).

For evaluating whether HRW could prevent the weight loss, mechanical allodynia, cold allodynia and GS deficits induced by CIS, male and female mice were pretreated with HRW, intraperitoneally administered at a concentration of 0.3 mM, 1 h before testing in a final volume of 10 mL/kg [38], followed by CIS or vehicle intraperitoneally administered 2 h later. A similar protocol was used for assessing whether HRW could prevent CIS-induced anxiety- and depressive-like behaviors.

### 2.4. Allodynia

Mechanical allodynia was measured as the hind paw withdrawal response to von Frey hair stimulation using the up-and-down method as described previously [49]. Mice were placed in methacrylate cylinders (20 cm high/9 cm diameter) in a grid bottom by which a series of von Frey hairs (0.4 to 3 g; North Coast Medical, Inc., San Jose, CA, USA) were perpendicularly applied to the plantar surface of each hind paw. A positive response was defined as a clear paw withdrawal or shaking. Whenever a positive response happened, the next lower hair was applied, and when a negative response occurred, the next higher hair was applied.

For cold allodynia, we used a cold plate analgesiometer (Ugo Basile, Varese, Italy) at 4 ± 0.5 °C and the number of elevations of each hind paw was noted for 5 min.

### 2.5. Forelimb Grip Strength Test

The grip strength was measured using a grip strength meter (Ugo Basile, Varese, Italy) according to [50]. The mice were allowed to grab the metal bar with both hind legs before the mouse’s tail was gently pulled back; the maximal force (g) was recorded automatically by the machine. The tests were performed three times for each animal at one-min intervals before CIS or VEH injection. This value was considered as 100% grip strength and was used as a reference for subsequent determinations. The force value was used to reflect muscle weakness.

### 2.6. Anxiety and Depressive Conducts

The elevated plus maze (EPM) with 4 arms, 2 closed by 15 cm high walls and 2 open, elevated at 45 cm from the floor was used to assess the anxiety-like behaviors [51]. Each arm was 5 cm wide and 35 cm long. Mice were placed in the center of the maze and permitted to explore it for 5 min. During this time, the animals were recorded with a digital camera and the number of entrances and proportion of time spent in the open arms were counted. 

The tail suspension test (TST) and the forced swimming test (FST) were used to assess the depressive-like behaviors. In the TST, mice were suspended at 35 cm from the floor by using adhesive tape to the tip of the tail and fixing it to a surface. The animals were filmed, and the immobility time was evaluated for 6 min [52]. In the FST, mice were put into transparent cylinders (height, 25 cm; radius, 10 cm) containing water (24 ± 2 °C) up to a 10 cm depth for 6 min. The time that the animals spent immobile during the last 4 min was recorded [53].

Animals were familiarized with the testing room for 1 h before beginning the tests.

### 2.7. Western Blot

For Western blot analysis, animals were euthanized by cervical dislocation at day 30 after the first injection of CIS or VEH. DRG from the ipsilateral lumbar section (L3 to L5) and PFC were extracted and preserved at −80 °C. 

Tissues were sonicated with cold RIPA buffer (Sigma-Aldrich, MO, USA). After solubilization for 1 h at 4 °C, the homogenizes were sonicated again for 10 s and centrifuged for 20 min at 700 g and 4 °C. Samples (60 µg total protein) were separated on a sodium dodecyl sulfate polyacrylamide gel (12%), transferred to polyvinylidene fluoride membranes for 120 min and blocked with phosphate-buffered saline plus Tween 20 or Tris-buffered saline plus Tween 20 + 5% of nonfat dry milk or bovine serum albumin for 75 min. Membranes were incubated overnight at 4 °C with specific primary antibodies (Table 1), followed by horseradish peroxidase-conjugated secondary antibody (GE Healthcare, Little Chalfont, UK). Proteins were detected with chemiluminescence reagents (ECL kit; GE Healthcare, Little Chalfont, UK), and densitometric analysis was performed using the Image-J program (version 1.8.0; National Institutes of Health, Bethesda, MD, USA).

### 2.8. Statistical Analyses

The results are expressed as the mean values ± standard error of the mean (SEM). A three-way repeated measures ANOVA with time, sex and treatment as the variation factors was used to assess the actions of the co-treatment with HRW on CIS-provoked allodynia, grip strength deficits and body weight loss. A two-way ANOVA was used to assess the influence of the co-treatment with HRW on the emotional behaviors caused by CIS. A one-way ANOVA plus Tukey test was used to analyze the impact of HRW treatment on the expression of several proteins. We used SPSS (version 28, IBM, Madrid, Spain) and GraphPad Prism 9.0 software (La Jolla, CA, USA). A *p* < 0.05 was declared as significant.

## 3. Results

### 3.1. The Prophylactic Treatment with HRW Inhibited the Mechanical and Cold Allodynia Generated by CIS in Male and Female Mice

On the evaluation of the effects of HRW on the mechanical allodynia provoked by CIS in both hind paws of male and female mice, the three-way ANOVA repeated measures test revealed a significant time effect (*p* < 0.001), a significant treatment effect (*p* < 0.001) and a significant time by treatment interaction (*p* < 0.001) in the von Frey test. 

**Figure 2:**
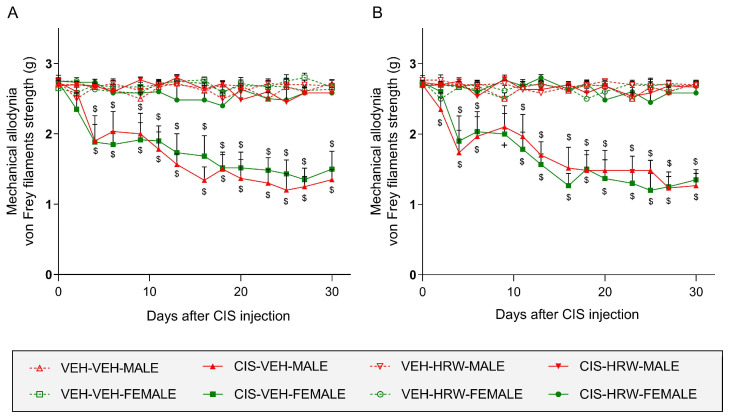
Treatment with HRW inhibited the mechanical allodynia caused by CIS in male and female mice. The actions produced by the prophylactic treatment with HRW on the mechanical allodynia observed in the left (**A**) and right (**B**) hind paws of CIS-injected male and female mice are represented. In both panels, for each day evaluated, $ represents significant differences vs. their respective VEH-VEH-, VEH-HRW- and CIS-HRW-treated animals and + vs. their respective VEH-HRW-treated mice (*p* < 0.05, one-way ANOVA plus Tukey test). Results are shown as mean values ± SEM; *n* = 8 animals.

The results indicated that the tactile allodynia caused by CIS was similarly manifested in both hind paws of male and female mice from day 4 to 30 after injection (*p* < 0.001 vs. their respective VEH-VEH treated mice; Figure 2A,B) and that the prophylactic treatment with HRW for 30 days avoided its development.

**Figure 3:**
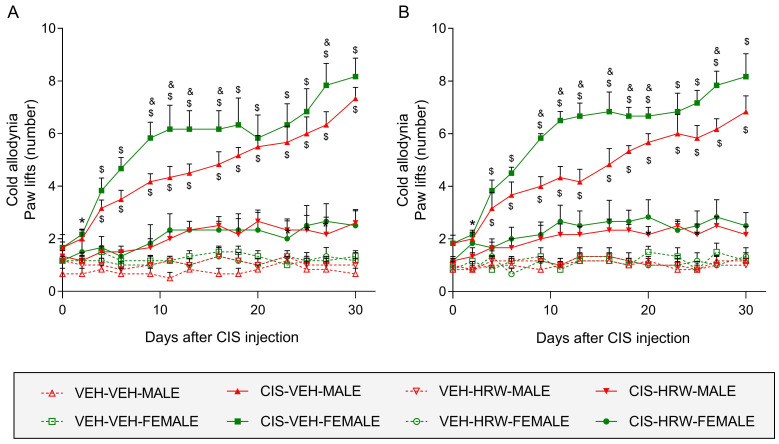
Treatment with HRW inhibited the cold allodynia caused by CIS in male and female mice. The actions produced by the prophylactic treatment with HRW on the cold allodynia observed in the left (**A**) and right (**B**) hind paws of CIS-injected male and female mice are represented. In both panels, for each day evaluated, * represents significant differences vs. their respective VEH-VEH- and VEH-HRW-treated animals, $ represents significant differences vs. their respective VEH-VEH-, VEH-HRW- and CIS-HRW-treated animals and & vs. CIS-VEH-treated male mice (*p* < 0.05, one-way ANOVA plus Tukey test). Results are shown as mean values ± SEM; *n* = 8 animals.

A significant time effect (*p* < 0.001), sex effect (*p* < 0.030), treatment effect (*p* < 0.001) and a time by treatment interaction (*p* < 0.001) was revealed in the cold plate test. Therefore, cold allodynia was manifested from day 4 to 30 after CIS injection but the number of the left paw lifts at days 9, 11, 13, 16 and 27 after CIS injection (*p* < 0.001; Figure 3A) and on days 9, 11, 13, 16, 18, 20 and 27 post CIS injection in the right paws of female mice were higher than in male animals (*p* < 0.001; Figure 3B). In both sexes and paws, cold allodynia was completely inhibited from day 4 to 30 of treatment with HRW.

In both tests and paws, treatment with HRW did not produce any effect in VEH-injected animals (Figure 2 and Figure 3).

### 3.2. The Effects of the Prophylactic Treatment with HRW on the Grip Strength Deficits and Body Weight Loss Produced by CIS in Male and Female Mice

**Figure 4:**
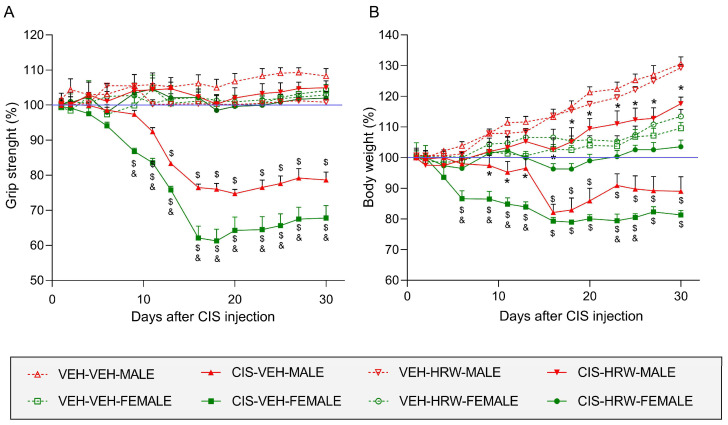
The effects of HRW on the grip strength deficits and body weight loss produced by CIS in male and female mice. The actions produced by the prophylactic treatment with HRW on the percentage of grip strength (**A**) and body weight (**B**) of CIS- and VEH-injected male and female mice are represented. In both graphs, for each day analyzed, $ represents significant differences vs. their respective VEH-VEH-, VEH-HRW- and CIS-HRW-treated animals, & vs. CIS-VEH-treated male mice and * vs. their respective VEH-VEH- and VEH-HRW-treated mice (*p* < 0.05, one-way ANOVA plus Tukey test). Results are shown as mean values ± SEM; *n* = 8 animals.

In this study, we also investigated the impact of sex on the grip strength deficits and body weight loss produced by CIS and its possible inhibition with the prophylactic treatment with HRW (Figure 4).

On the grip strengths of hind paws from male and female mice, the three-way repeated measures ANOVA showed significant effects for time and treatment (*p* < 0.001) and the interactions between time and sex, time and treatment and sex and treatment (*p* < 0.009) and among time, treatment and sex (*p* < 0.006). Our results demonstrated grip strength deficits caused by CIS in female mice from day 9 to 30 after its injection and in male animals from day 13 to 30 after its injection (*p* < 0.001; one-way ANOVA compared with their respective VEH-VEH-injected mice) (Figure 4A). In addition, the reduction in the grip strength caused by CIS from day 9 to 30 after injection was higher in female than in male mice (*p* < 0.001; one way ANOVA). Interestingly, the co-administration of HRW avoided the grip strength deficits caused by CIS in both sexes.

Regarding body weight, significant effects for time, sex and treatment (*p* < 0.004) and interactions between time and sex, time and treatment and among time, treatment and sex (*p* < 0.001) were revealed by a three-way repeated measures ANOVA. The administration of CIS provoked a body weight loss from day 6 to 30 after its injection in female mice and from day 16 to 30 in male animals (*p* < 0.001; one-way ANOVA compared with their respective VEH-VEH-injected mice; Figure 4B). In concordance with the grip strength, the weight loss provoked by CIS started earlier in female than in male mice. Moreover, the weight loss provoked by CIS was higher in female than in male mice from day 6 to 13 and from day 23 to 25 after CIS injection (*p* < 0.001; one-way ANOVA) and was completely reversed in female mice but only partially in male mice from day 16 to 30 of treatment (*p* < 0.001; one-way ANOVA compared with their respective VEH-VEH- and VEH-HRW-treated mice).

In all cases, treatment with HRW did not produce any effect on VEH-injected animals (Figure 4).

### 3.3. Treatment with HRW Inhibited the Emotional Disorders Causes by CIS

**Figure 5:**
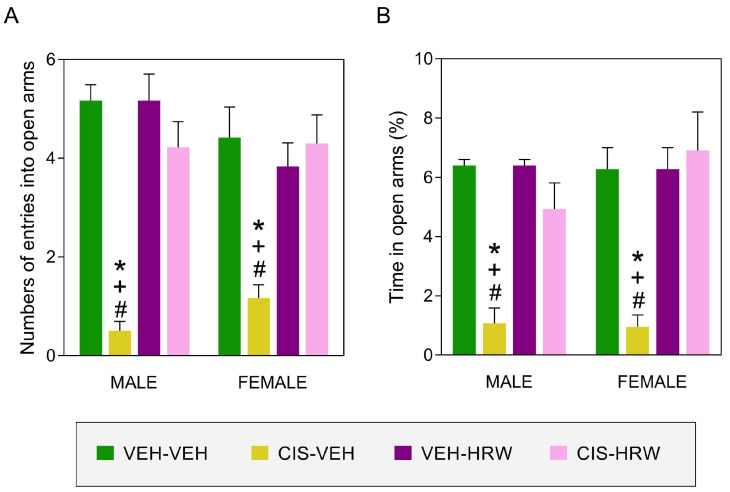
The effects of treatment with HRW on the anxiety-like comportments related to CIS injection. The impact of the prophylactic administration of HRW for 30 consecutive days on the anxiety-like behaviors provoked by CIS in male and female mice are represented. The number of entrances into the open arms (**A**) and the percentage of time spent in them (**B**) are shown. In both graphs, the symbols denote significant changes vs. their respective * VEH-VEH-, + VEH-HRW- or # CIS-HRW-treated animals (*p* < 0.05, one-way ANOVA plus Tukey test). Results are presented as mean values ± SEM; *n* = 8 animals.

Our data confirmed the anxiety-like behaviors (Figure 5) produced by CIS and revealed that non-sex differences were detected in this affective disorder. Indeed, the significant effects of CIS and treatment (*p* < 0.003) and their interaction (*p* < 0.004) on the number of entrances (Figure 5A) and time spent in the open arms for both male and female mice (Figure 5B) were revealed by the two-way ANOVA. For both sexes, the decreased number of entries to the open arms and the low amount of time spend in them suggested an anxiety-like status that was completely reversed by the administration of HRW for 30 consecutive days (*p* < 0.001; one-way ANOVA).

**Figure 6:**
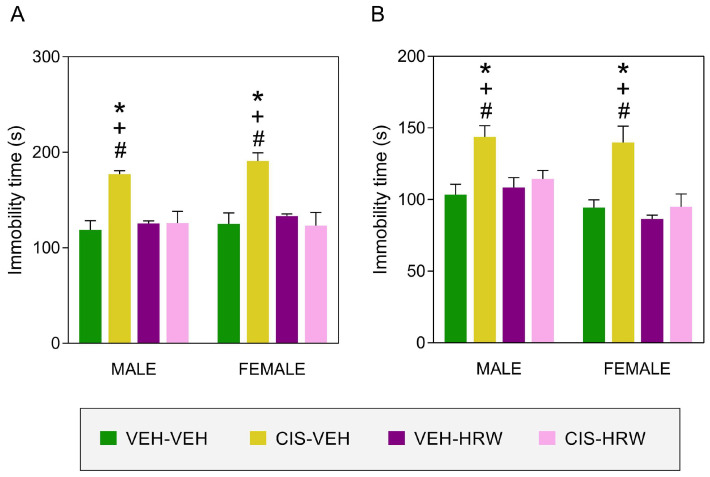
The antidepressant properties of HRW in male and female CIS-injected mice. Effects of the prophylactic treatment with HRW for 30 consecutive days on the depressive-like comportments related to CIS injection. The time that animals remained immobile (s) in the TST (**A**) and FST (**B**) are displayed. In both graphs, the symbols denote significant changes vs. their respective * VEH-VEH-, + VEH-HRW- and # CIS-HRW-treated mice (*p* < 0.05, one-way ANOVA plus Tukey test). Results are presented as mean values ± SEM; *n* = 8 animals.

Our data also demonstrated an enhancement of the immobility time of CIS-injected male and female animals in the TST (Figure 6A) and FST (Figure 6B) (*p* < 0.001; one-way ANOVA compared with their respective VEH-VEH-treated animals) suggesting a depressive-like status that was completely reversed by the administration of HRW. Thus, significant effects of CIS and treatment (*p* < 0.010) and their interaction (*p* < 0.010) were revealed by the two-way ANOVA in the TST and FST. Non-significant differences between the sexes were identified.

### 3.4. Effects of HRW on the Inflammatory and Oxidative Reactions Provoked by CIS in the DRG and PFC

**Figure 7:**
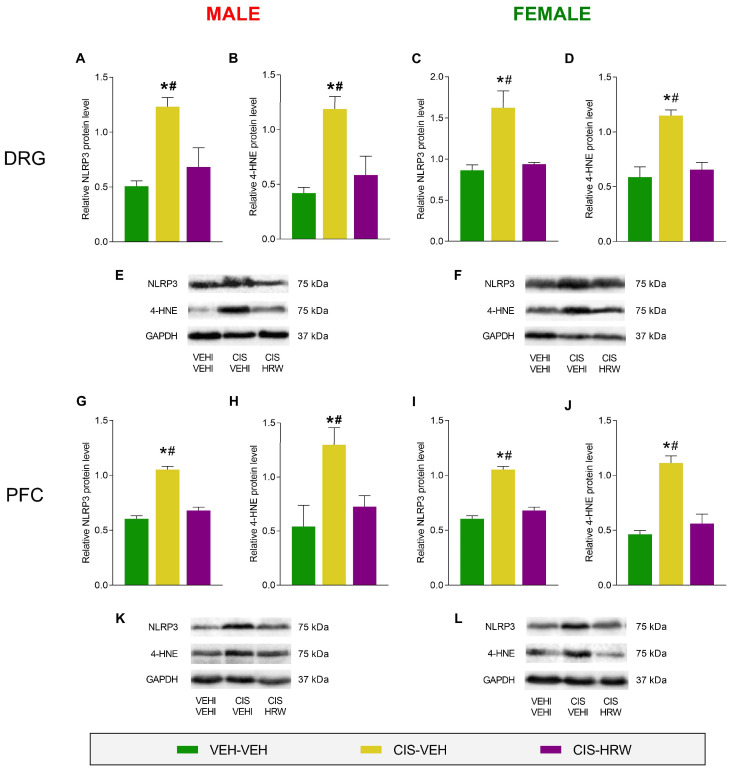
The impact of HRW treatment on the protein levels of NLRP3 and 4-HNE in the DRG and PFC of male and female CIS-injected mice. The protein levels of NLRP3 in the DRG (**A**,**C**) and PFC (**G**,**I**) and those of 4-HNE in the DRG (**B**,**D**) and PFC (**H**,**J**) from male and female CIS-injected mice are shown. Control animals treated with VEH plus VEH are also represented. Representative blots of NLRP3, 4-HNE and GAPDH (**E**,**F**,**K**,**L**) from the DRG and PFC of male and female mice are also shown. In all graphs, the symbols denote significant changes vs. their respective * VEH-VEH- or # CIS-HRW-treated animals (*p* < 0.05, one-way ANOVA plus Tukey test). Data are expressed as mean values ± SEM; *n* = 3 samples per group.

Taking in to account the important contribution of inflammation and oxidative stress in the progress of CIS-induced neuropathy and/or in the physical and mental incapacities associated with it, the influence of the HRW treatment on the protein levels of the NLRP3 inflammasome and the oxidative stress marker 4-HNE in the DRG and PFC of CIS-injected male and female mice were evaluated (Figure 7).

Peripheral and central inflammatory reactions triggered by CIS were demonstrated by the enhanced levels of NLRP3 observed in the DRG (*p* < 0.010, one-way ANOVA; Figure 7A,C) and PFC (*p* < 0.002, one-way ANOVA; Figure 7G,I) from male and female mice compared with their respective VEH-VEH-treated mice. Interestingly, treatment with HRW normalized this inflammatory reaction in tissues from both male and female mice. Our results also showed an overexpression of 4-HNE in the DRG (*p* < 0.010, one-way ANOVA; Figure 7B,D) and PFC (*p* < 0.020, one-way ANOVA; Figure 7H,J) from male and female CIS-injected animals, revealing the oxidative stress caused by this chemotherapeutic agent in the central and peripheral nervous system, which was completely inhibited by HRW treatment. These findings showed the anti-inflammatory and antioxidant actions produced by the prophylactic treatment with HRW in CIS-injected male and female mice.

**Figure 8:**
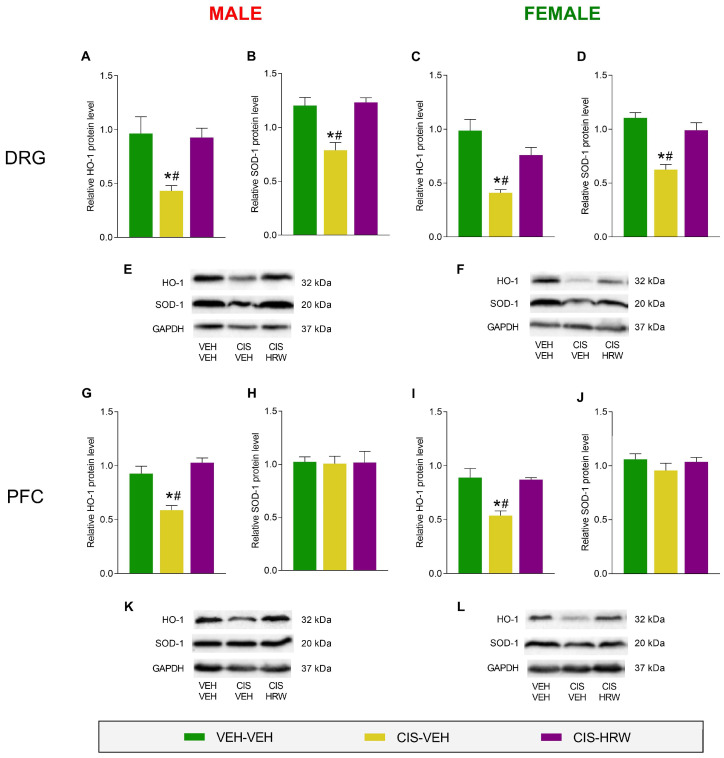
The impact of HRW treatment on the protein levels of HO-1 and SOD-1 in the DRG and PFC of male and female CIS-injected mice. The protein levels of HO-1 in the DRG (**A**,**C**) and PFC (**G**,**I**) and those of SOD-1 in the DRG (**B**,**D**) and PFC (**H**,**J**) of male and female CIS-injected mice are represented. Control animals treated with VEH plus VEH are also represented. Representative blots of HO-1, SOD-1 and GAPDH (**E**,**F**,**K**,**L**) from the DRG and PFC of male and female mice are also presented. In all graphs, the symbols denote significant changes vs their respective * VEH-VEH- or # CIS-HRW-treated animals (*p* < 0.05, one-way ANOVA plus Tukey test). Data are expressed as mean values ± SEM; *n* = 3 samples per group.

We also analyzed the effectiveness of HRW treatment in activating the endogenous antioxidant system as a mechanism of defense to the inflammatory and oxidative reactions initiated by CIS in the central and peripheral nervous systems of male and female mice. Our data showed decreased expression of HO-1 in the DRG (*p* < 0.022, one-way ANOVA; Figure 8A,C) and PFC (*p* < 0.007, one-way ANOVA; Figure 8G,I) of male and female mice injected with CIS compared with their respective VEH-VEH-treated animals. Remarkably, in both sexes and tissues evaluated, the downregulation of HO-1 was normalized by HRW treatment. CIS also decreased the protein levels of SOD-1 in the DRG of male (*p* < 0.005, one-way ANOVA; Figure 8B) and female mice (*p* < 0.002, one-way ANOVA; Figure 8D) vs. their respective VEH-VEH-treated animals but not in the PFC of male (*p* < 0.990, one-way ANOVA; Figure 8H) or female animals (*p* < 0.964, one-way ANOVA; Figure 8J). In both sexes, the downregulation of SOD-1 in the DRG was inhibited by the administration of HRW.

## 4. Discussion

Our results demonstrated that CIS-based chemotherapy caused mechanical and cold allodynia, grip strength deficits, body weight loss and anxiodepressive-like behaviors in male and female mice. Sex differences in the progression of cold allodynia, grip strength deficits and body weight loss provoked by CIS were observed. Prophylactic treatment with HRW prevented the onset of allodynia, functional dysfunctions and mood impairments resulting from CIS in both sexes. These effects could be mediated by modulating the inflammatory and oxidative reactions provoked by CIS in the DRG and PFC.

In accordance with other works carried out in male [15,45,54,55] or female mice [46,56], our results demonstrated that CIS produced similar persistent tactile allodynia, up to 30 days after its first injection, in both hind paws of male and female mice. These results revealed that no differences in CIS-induced mechanical allodynia were observed between male and female mice. These findings are consistent with the non-sex differences reported by other authors in CIS- [4,14,57] and vincristine-provoked tactile allodynia [22]. However, in contrast, with the sex variations demonstrated by Woller et al. (2015) [15], which showed that the tactile allodynia caused by CIS disappeared 20 days after its first injection in female but not in male mice. These discrepancies might be attributed to the different doses and pattern of CIS administration used in these studies. Therefore, whereas in our experiments animals received a daily dose of 2.8 mg/kg for five days followed by five days of rest and a second round of five doses, in another experiment [15], the animals were given 2.3 mg/kg of CIS every other day over the course of 2 weeks. 

Our data further revealed that CIS induced cold allodynia in both hind paws of male and female mice from day 4 to 30 after injection. However, unlike tactile allodynia, cold allodynia progresses differently between the sexes. Indeed, the number of paw lifts on days 9 to 16 and at day 27 after CIS injection were higher in female than in male mice. In contrast, Naji-Esfahani et al. (2016) [14] revealed that CIS injection evoked cold allodynia in male and female mice but no significant differences between the sexes were observed from day 7 to 15 after CIS injection. The dose and the administration guidelines by which CIS was injected might be the main reason for these divergences. In the experiments performed by Naji-Esfahani et al. (2016) [14], the animals only received one daily dose of 1 mg/kg of CIS for seven days, which might not be enough for establishing differences between the sexes. Our findings also demonstrated that the prophylactic treatment with HRW prevented the tactile and cold allodynia generated by CIS in both hind paws and both sexes. These results are consistent with the analgesic actions produced by the preventive treatment with HRW or hydrogen-rich saline (HRS) in animals with neuropathic pain caused by nerve injury [58,59,60,61] and further revealed the antiallodynic properties of HRW in CIS-injected mice. Additionally, our data coincide with the prevention of the development of mechanical allodynia caused by CIS in mice prophylactically treated with two peptide antagonists for kinin B1 and B2 receptors [55].

It is well acknowledged that CIS-based chemotherapy is also associated with other side effects including muscle wasting and body weight loss [29,30]. Our results corroborated these effects by demonstrating that CIS injection produced grip strength deficits in the hind paws of both sexes. These data agreed with the decreased muscle strength observed in male [31,32,33,62,63] and female animals [64] treated with CIS. Nonetheless, our findings further proved that female mice have lower grip strength in comparison with male mice from day 9 to 30 of CIS injection, thus revealing the impact of sex in this side effect produced by CIS in mice. 

Considering that the body weight loss in cancer patients treated with CIS is mostly due to muscle wasting [10], the body weight loss caused by CIS was also evaluated in this study. As expected, a significant body weight loss was demonstrated in male and female CIS-injected mice starting at 6 or 9 days after CIS injection and lasting until 30 days. Similarly, other studies also demonstrated that the body weight of CIS-injected male [31,32,33,63] and female animals [46,64] was significantly lower than their respective controls. Our data further showed that the weight loss provoked by CIS was higher in female than in male mice from day 6 to 13 and at days 23 and 25 after its injection, identifying sex differences in this aspect. Our experiments also proved that the co-administration of HRW prevented the CIS-induced muscle strength deficits and body weight loss in both sexes. Nonetheless, considering that female mice injected with CIS exhibit a higher percentage of grip strength deficit and body weight loss compared with male mice injected with CIS, it appears that the inhibitory effects induced by HRW are more prominent in female mice. The administration of HRS also reduces body weight loss after morphine withdrawal in mice [65]. The inhibition of the decreased muscle strength and the loss of body weight provoked by CIS through the co-administration with HRW is key for avoiding the reduction of these physical functions provoked by this antineoplastic agent in cancer patients, which negatively affects their quality of life [66]. 

Inflammation and disruption of redox homeostasis were considered two significant contributors of the CIS-evoked neuropathic pain [25]. Thus, anti-inflammatory and antioxidant drugs attenuated the nociceptive responses caused by CIS [27,67]. In line with this, our results demonstrated increased levels of the inflammasome NLRP3 and the oxidative marker 4-HNE, accompanied by the decreased expression of HO-1 and SOD-1 in the DRG of CIS-injected male and female mice. Treatment with HRW normalized these inflammatory and oxidative reactions caused by CIS in the DRG, revealing the potent anti-inflammatory and antioxidant actions of this treatment, as previously demonstrated in paclitaxel-injected mice [37]. H_2_ inhibited the neuropathic pain caused by nerve lesions by reversing the overexpression of proinflammatory cytokines and 4-HNE [58]. In line with this, a recent study demonstrated that the treatment with several HO-1 inducers also inhibited neuropathic pain by inhibiting the up- and downregulation of NLRP3 and HO-1, respectively, in the DRG [68]. Other works further revealed that the analgesic effects of H_2_ were reversed by an HO-1 inhibitor and enhanced by its co-administration with carbon monoxide donors [60]. These results suggest that the maintenance of an equilibrium between the NLRP3 and the antioxidant system is key for the alleviation of CIS-induced neuropathy produced by HRW. Supporting this theory, a negative feedback system between the NLRP3 and Nrf2 pathways has been demonstrated. Therefore, activation of the Nrf2 signaling can inhibit the NLRP3 activation, thus reducing ROS production [69], and Nrf2 can also attenuate several inflammatory mediators derived from NF-κB, resulting in downregulation of the NLRP3 inflammasome activity [70]. 

The anxiety- and depressive-like behaviors observed in male and female mice receiving CIS agreed with the anxiodepressive-like behaviors produced by this antineoplastic agent in humans [10] and with other experimental results mainly performed in male animals [28,32,62,71]. Our results further showed that these emotional alterations were similarly displayed in both sexes. Thus, in both male and female mice, this chemotherapeutic agent similarly diminished the number of entries and the time spent in the open arms in the EPM and increased the time that mice remained immobile in the TST and FST. Our data also proved that the administration of HRW avoided these affective disorders, thus revealing the anxiolytic and antidepressant actions produced by the prophylactic treatment with HRW in CIS-injected mice. In agreement, this treatment likewise inhibited the emotional deficits linked with inflammatory and neuropathic pain provoked by nerve injury or paclitaxel [37,38,39]. HRW also prevents the depressive-like comportments generated by mild chronic unpredictable stress [72] and attenuates the anxiety caused by opioid withdrawal in mice [65]. In addition, considering that conventional antidepressants exhibit significant adverse effects whereas HRW does not exhibit demonstrable secondary effects [35], the preventive treatment with HRW might be considered as an alternative for the management of affective disorders associated with CIS chemotherapy.

One of the multiple processes involved in the development of the mood disorders related to neuropathic pain is oxidative stress. CIS administration causes overproduction of ROS and an imbalance between the oxidant and antioxidant protein levels, leading to an insufficient quantity of antioxidants to stabilize ROS production in tissues [73]. Therefore, the increased expression of 4-HNE and the diminished activity of the antioxidant enzyme HO-1 observed in the PFC corroborated the oxidative effects caused by CIS in the central nervous system. These findings are in harmony with the enhanced TBARS values and the decreased levels of other antioxidant enzymes such as catalase in the PFC [28] and hippocampus [74,75] from CIS-injected animals. Our data did not support the diminished expression of SOD-1 stimulated by CIS in the PFC of rodents [28,74,75]. These differences might be accredited to the different times for evaluating these responses (10 days vs. 30 after CIS injection). 

Our study also revealed that the inflammatory and oxidative alterations caused by CIS in the PFC were regulated by the administration of HRW through normalizing the upregulation of NLRP3 and 4-HNE and the downregulation of HO-1 in this brain area. Considering the significance of inflammation and oxidative stress in the development of affective behaviors, their inhibition with HRW in the PFC, an area involved in the control of emotions, suggested that the anti-inflammatory and antioxidant actions of this treatment might be implicated in the modulation of the emotive behaviors. In agreement with this, the administration of HRW also diminished the brain activation of the NLRP3 inflammasome and the expression of its downstream inflammatory molecules in a preclinical model of Alzheimer’s disease [76] and HRS reduced the levels of NLRP3 in the cortex of rats with subarachnoid hemorrhage [77]. Likewise, other treatments, for example edaravone, also modulated the mood deficits caused by CIS via inhibiting oxidative stress and inflammation in the rat hippocampus [78]. These data support the hypothesis that HRW treatment might also inhibit the anxiety- and depressive-like behaviors caused by CIS by blocking the inflammasome NLRP3 and activating the defense endogenous antioxidant system in the PFC. Finally, considering the few side effects induced by HRW [79], it might be proposed as a safe and effective therapy against CIS-induced neuropathic pain.

A limitation of this study is that we have only evaluated the effects produced by the intraperitoneal administration of HRW; it might be interesting to study the actions produced by the oral administration of HRW on the allodynia, body weight loss, grip strength deficits and the emotional disorders caused by CIS.

## 5. Conclusions

This study reveals that the prophylactic treatment with HRW prevents the allodynia, muscle strength deficits, body weight loss and affective disorders caused by CIS. This treatment stabilized the inflammatory and oxidative responses stimulated by CIS in the DRG and PFC. Our findings suggest that HRW might be a safe treatment for the management of CIS-induced neuropathy and the associated dysfunctional and affective deficits that would greatly increase the quality of life of patients receiving this kind of chemotherapy.

## Figures and Tables

**Table 1:** Primary antibodies used in this study.

Antibody	Dilution	Purchased
NLRP3	1:200	Adipogen Life Sciences, Epalinges, Switzerland
4-HNE	1:200	Abcam, Cambridge, UK
HO-1	1:100	Enzo Life Science, New York, NY, USA
SOD-1	1:150	Novus Biologic, Littleton, CO, USA
GAPDH	1:5000	Merck, Billerica, MA, USA

## Data Availability

Data is contained within the article.
